# Genome Sequence of *Oceanimonas doudoroffii* ATCC 27123^T^

**DOI:** 10.1128/genomeA.00996-17

**Published:** 2017-09-07

**Authors:** Marc A. Brennan, Ariel M. Trachtenberg, William D. McClelland, Kyle S. MacLea

**Affiliations:** a Biotechnology Program, University of New Hampshire, Manchester, New Hampshire, USA; b Biology Program, University of New Hampshire, Manchester, New Hampshire, USA; c Department of Life Sciences, University of New Hampshire, Manchester, New Hampshire, USA

## Abstract

*Oceanimonas doudoroffii* ATCC 27123^T^ is an obligately aerobic Gram-negative rod of the class *Gammaproteobacteria*. It was first isolated from surface seawater off the coast of Oahu, HI, USA, in 1972. The predicted genome size is 3,832,938 bp (G+C content, 60.03%), which contains 3,524 predicted coding sequences.

## GENOME ANNOUNCEMENT

*Oceanimonas doudoroffii* strain 70 (ATCC 27123^T^) is a Gram-negative obligately aerobic straight-rod marine bacterium that is motile by means of 1 to 3 flagella at each pole (1, 2). This gammaproteobacterial species was first isolated from surface seawater off the coast of Oahu, HI, USA, by Baumann et al. in 1972, along with other nonfermentative marine bacteria then thought to be pseudomonads (1). A wholesale reclassification based on DNA-rRNA hybridization methods (3) suggested that about two-thirds of *Pseudomonas* species were misclassified. Brown et al. placed *Pseudomonas doudoroffii* ATCC 27123^T^ in a new genus, *Oceanomonas* (4), with spelling later corrected to *Oceanimonas* (5). *Oceanimonas doudoroffii* strain 70 (ATCC 27123) was designated the type strain for the genus, and strain GB6 was given the name *Oceanimonas baumannii* ATCC 700832 as the type strain for its species (4). In 2005, a third novel *Oceanimonas* species was discovered, *Oceanimonas smirnovii*, with strain ATCC BAA-899 (6).

*O. doudoroffii* is a chemoorganotroph that can grow at temperatures from 10 to 40°C (4) and requires seawater/sodium ions for growth (2), although only up to 5% NaCl (6). *O. doudoroffii* is catalase and oxidase positive, accumulates polyhydroxybutyrate (PHB), and is capable of growing on benzoate or *p-*hydroxybenzoate to degrade catechol or protocatechuate by means of *o-*cleavage (1).

*O. doudoroffii* ATCC 27123^T^ was purchased from the ATCC (Manassas, VA, USA) in freeze-dried form, rehydrated, and grown in marine broth or agar (ATCC medium 2216) at 26°C for 24 h at atmospheric pressure. After successful growth, a single colony was cultured in log phase, and genomic DNA (gDNA) was isolated using the Genomic-tip 500/G kit (Qiagen, Valencia, CA, USA). gDNA was fragmented, ligated with adapters using the Nextera DNA library prep kit (Illumina, San Diego, CA, USA), and sequenced with 250-bp paired-end reads on an Illumina HiSeq 2500 platform at the Hubbard Center for Genome Studies (Durham, NH, USA). Trimmomatic was used for computational removal of adapter sequences and small fragments (7).

The draft genome of *O. doudoroffii* was assembled using SPAdes version 3.8.0 (8) into 19 final trimmed contigs. These contigs had a total length of 3,832,938 bp and an average coverage of 66.3× (9). The *N*_50_ value and largest contig found was 2,085,234 bp, with a G+C% of 59.79% for this single large contig. The G+C results for the genome and largest contig are in close agreement with previous reports of G+C contents by CsCl gradient, 59.7% (1), 59% (6), and 58 to 60% (2), with less agreement with the previous report by the high-performance liquid chromatography (HPLC) method, at 54% (4).

The NCBI Prokaryotic Genome Annotation Pipeline (PGAP) process (9) was used to find and assign names to the genes in the genome. PGAP labeled a total of 3,627 genes, 3,540 coding sequences (CDSs), 87 RNA genes, 16 pseudogenes, and 1 clustered regularly interspaced short palindromic repeat (CRISPR) array. Notably, a copy of the 16S rRNA was not found, although six copies of the 5S and three copies of the 23S rRNA were identified.

### Accession number(s).

This whole-genome shotgun project has been deposited at DDBJ/ENA/GenBank under the accession no. NBIM00000000. The version described in this paper is version NBIM01000000.
